# Protocol for Monitoring Postpartum Hypertension Outcomes via a Smartphone App (MOMitor): Ecological Momentary Assessment

**DOI:** 10.2196/91392

**Published:** 2026-05-22

**Authors:** Chidimma Doris Azubuike, Jungjun Bae, Deepthi S Varma, Tony Wen, Kay Roussos-Ross, Amie Goodin

**Affiliations:** 1 Department of Pharmaceutical Outcomes and Policy, University of Florida, DSIT Building, 6th Floor, 1889 Museum Road, Gainesville, FL, 32611-0496, United States, 1 3522948829

**Keywords:** hypertension, postpartum, maternal health, digital health, pregnancy

## Abstract

**Background:**

Postpartum hypertension defined as elevated blood pressure after childbirth, affects approximately 20% of women after delivery. Digital health interventions that include remote monitoring therefore, present an important opportunity to facilitate regular blood pressure assessment in this high-risk population.

**Objective:**

The primary objective is to assess within- and between-person changes in hypertension status over the first 6 weeks postpartum using a smartphone-based ecological momentary assessment protocol. The secondary objective is to characterize the trajectory of hypertension indicators across the 6-week period and to compare hypertension screening results from week one and week six within participants.

**Methods:**

This study uses a time-series design using data from prospectively enrolled patients within a large health system. Patients eligible for recruitment were women aged 18 years or older who spoke English, owned a smartphone, had a liveborn neonate, and were willing to download an app and respond to survey questions within the app. Throughout the study period, all participants received hypertension screening items that asked whether their blood pressure was greater than 140/90 mm Hg and whether they felt dizzy or lightheaded, short of breath, or had a severe headache. A subset of participants were additionally asked to report their blood pressure readings. We estimate within- and between-participant effects using a generalized linear mixed model for a positive hypertension screen during weeks one through six. The model includes both fixed and random effects, controlling for age, race, marital status, education, insurance status, mode of delivery, total number of pregnancies, and history of diabetes mellitus.

**Results:**

The study was funded July 2021 and data collection was initiated in November 2021. Data collection for this study, per protocol, was completed in June 2025, though other participants continue to be enrolled in the ongoing parent study. Data analysis is expected to be completed by Spring 2026 with publication planned for Summer 2026.

**Conclusions:**

The study findings will provide insight into trajectories of hypertension outcomes in postpartum women and the utility for smartphone app-based remote blood pressure monitoring among postpartum women whose blood pressure was regularly tracked. Findings will provide evidence to support whether widespread implementation of this digital health surveillance approach to improve outcomes in maternal health, especially in the postpartum period, is clinically warranted.

## Introduction

Postpartum hypertension (PPHTN), defined as elevated blood pressure after childbirth, affects approximately 20% of women after delivery. Its incidence has been increasing in parallel with rising maternal age and higher rates of obesity among pregnant individuals [1]. PPHTN can occur de novo or in women with a history of hypertension. However, the risk of postpartum hypertension is known to be higher among women who have experienced hypertensive disorders of pregnancy (HDP), such as chronic hypertension, preeclampsia, eclampsia, and gestational hypertension [2,3]. While HDP is known to be a leading cause of morbidity and mortality [2,4], PPHTN is particularly concerning, contributing to over 70% of deaths related to hypertension during the postpartum period [5].

HDPs are common complications in pregnancy and predispose individuals to stroke, kidney disease, coronary heart disease, later development of chronic hypertension [4,6]. Women with HDPs face nearly a three-fold higher risk of cardiovascular-related complications compared to non-hypertensive women [7]. Given these elevated risks, the American Heart Association (AHA) and the American College of Obstetricians and Gynecologists (ACOG) recognize the postpartum period as a critical window for blood pressure management and cardiovascular risk assessment to improve long-term heart health [8]. Accordingly, ACOG recommends that individuals with HDPs receive an early postpartum evaluation within 7‐10 days of delivery, in addition to the routine 6-week postpartum visit [9]. However, attendance to postpartum visits is low, with nearly half of women missing their 6-week postpartum visit [10], due to systemic challenges with limited parental leave from work, childcare barriers, transportation barriers, scheduling barriers, and limited support networks [11-14]. Insurance plans in the United States may also not be required to fully cover additional visits outside of the routine postpartum visit (at 6 wk) for uncomplicated pregnancies [15], meaning that most women have limited or no contact with their health care providers in the weeks between hospital delivery discharge and the routine 6-week postpartum visit [16,17]. Although blood pressure in hypertensive pregnancies is generally expected to normalize by day 7 postpartum, pregnancy-related hypertension may persist throughout the postpartum period, extending beyond the early postpartum period [18]. Despite these recommendations, postpartum follow-up visits remain suboptimal [9,19]. Less than 50% of women with HDP attend at least one postpartum visit in the six weeks following delivery [9]. Digital health interventions that include remote monitoring therefore, present an important opportunity to facilitate regular blood pressure assessment in this high-risk population at a time when risk is elevated and when health care system engagement and communication with clinicians is limited.

Several studies have investigated strategies to enhance postpartum hypertension monitoring beyond scheduled clinic visits. One randomized clinical trial (RCT) used a Bluetooth-enabled remote device for daily blood pressure measurements over 6 weeks postpartum and observed systolic and diastolic pressures peaking around days 9‐12 before declining [2]. This study, however, included only women who developed de novo postpartum hypertension. RCTs among women with previously diagnosed gestational diabetes and preeclampsia showed improved diastolic BP readings [20]. Racial differences in PPHTN resolution have also been documented, with non-Black women showing faster resolution than Black women [21]. Additionally, a specialized postpartum hypertension clinic was established to improve detection and management among patients with severe HDPs [19]. While the clinic identified numerous untreated hypertensive cases missed by standard care, the median time to the first visit was 13 weeks postpartum, highlighting a significant gap in early monitoring. Further work by Socrates et al evaluated the acceptability of two telemonitoring strategies (a smartphone app and a programmed Excel spreadsheet) [22]. Both were well-received, though most participants preferred the smartphone-based option.

Research on hypertension trajectories among women at high risk for postpartum hypertension using digital health remote monitoring tools remains limited. As hypertension can develop or emerge quickly in this population, and in-person clinical assessment opportunities are limited in the 6-week period between delivery hospitalization discharge and the 6-week routine follow up visit, this population is ideally situated to potentially benefit from remote hypertension monitoring using technologies that allow for patient-reported data to be communicated with their healthcare providers. A useful strategy for collection of real-time data using repeated measures is Ecological Momentary Assessment (EMA) [23], which has been deployed to investigate trajectories of blood pressure as well as other physical and mental health symptoms that are subject to develop or change within a defined time period [24,25]. This study evaluates the course of hypertension status among women who underwent frequent blood pressure monitoring via a smartphone app by leveraging an EMA approach in combination with clinical team follow-up when positive screening for hypertension is detected from a participant. The primary objective is to assess within- and between-person changes in hypertension status over the first 6 weeks postpartum using a smartphone-based ecological momentary assessment protocol. The secondary objective is to characterize the trajectory of hypertension indicators across the 6-week period and to compare hypertension screening results from week one and week six within participants. The findings will enhance understanding of how digital health-based remote monitoring influences hypertension outcomes among postpartum women engaged in a smartphone-based intervention.

## Methods

### Study Design

This study uses a time series design utilizing data from prospectively enrolled patients from a large health-system. We use an ecological momentary assessment (EMA) technique with a series of repeated measures to collect data from participants for the first six weeks postpartum following discharge from the delivery hospitalization.

### Ethical Considerations

This study was granted ethical approval by the University of Florida IRB (UF IRB 202101618). Participants provided written informed consent prior to enrolling and were allowed to opt out at any point in the study. All identifiable features of the research participants were omitted in this study. Participants received a $30 gift card as compensation for their participation.

### Study Population and Recruitment

Participants were recruited in the MOMitor app study from the postpartum inpatient unit of a quaternary hospital from November 2021 through June 2025. Patients eligible for recruitment were women 18 years or older, who spoke English, owned a smartphone, had a liveborn neonate, were willing to download an app, and respond to survey questions in the app. Randomization was not used for enrollment as all eligible patients were flagged for contact by study personnel for recruitment and offered the opportunity for study participation (ie, enrollment was via a census strategy rather than sampling of eligible patients). Women were approached during their delivery hospitalization on nonholiday weekdays and offered study enrollment, with consent obtained if they agreed. Patients were eligible for the hypertension arm of the study if they had a current or any history of hypertension diagnosis, including pre-eclampsia, eclampsia, and gestational hypertension, or had a current or any history of elevated blood pressure reading (≥140/90). In this study, we focus on patients in the hypertension arm of the study.

Participants who were classified as inactive or withdrawn contributed to within- and between-participant analysis if they completed at least one hypertension screening at any point in the study period. Patients are censored at the time of their withdrawal or inactivity. Inactive participants are defined as having more than three consecutive noncompleted survey instances, accompanied by at least three unsuccessful contact attempts via phone call, but inactive participants are still included in the analysis. Participants who requested discontinuation, “opt out” of receiving notifications and reminders to submit responses to hypertension screening questions were classified as withdrawn.

### Data Collection

**Figure 1.**
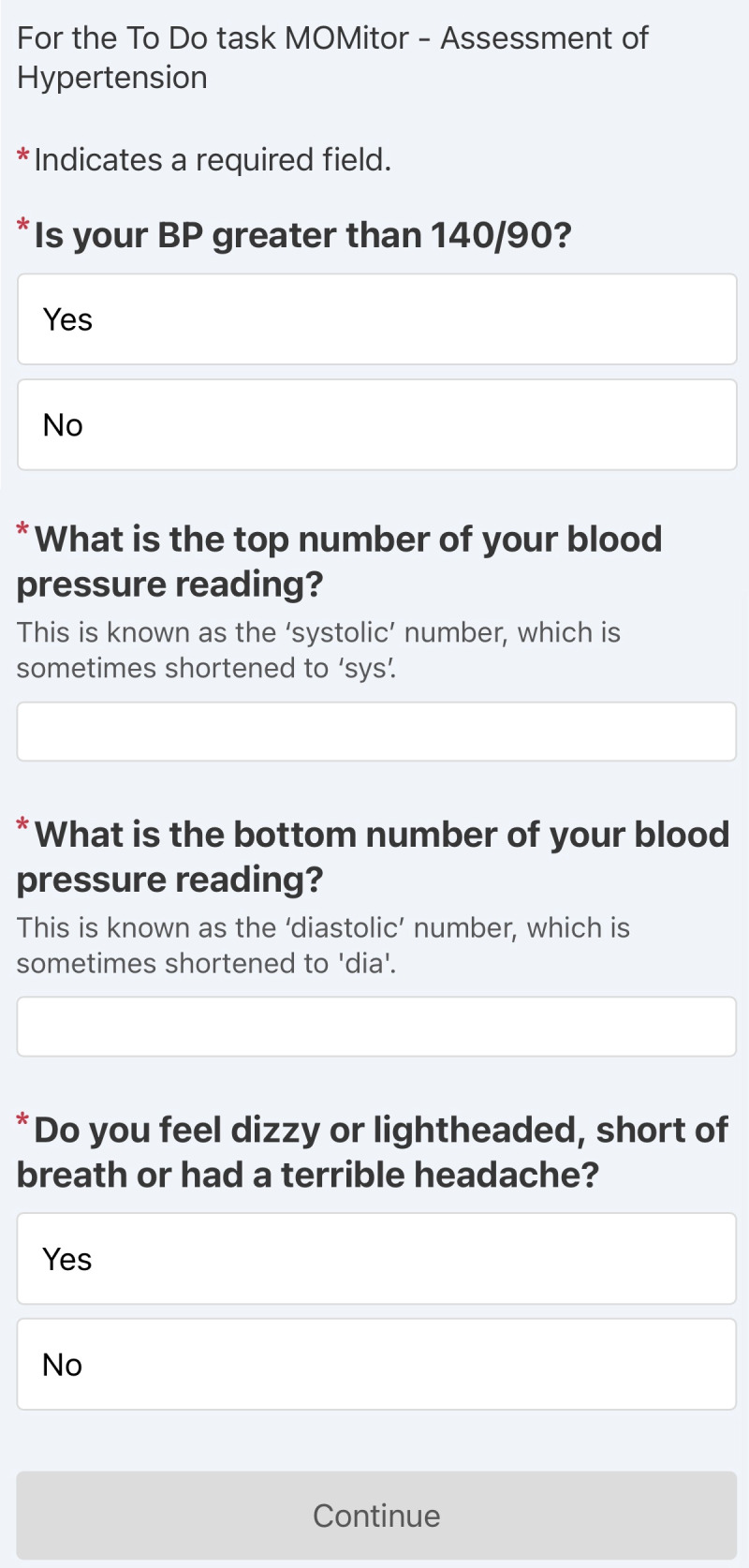
Snapshot of the hypertension screening questions as viewed in the app.

Baseline information was obtained through self-report at enrollment and entered into REDCap (Research Electronic Data Capture) by the research coordinators. Baseline clinical information was further validated using electronic health record chart review including *ICD* (International Statistical Classification of Diseases) codes and clinical notes review with a 1-year lookback period. Participant hypertension status and symptoms are recorded by the patient in real time in the MOMitor app. The app was developed by the research team to monitor a variety of physical and mental health symptoms experienced by women during pregnancy and postpartum. This study exclusively examines data from women recruited into the hypertension monitoring arm of the parent MOMitor app study. More information about the MOMitor app is available on the originating health-system’s website [26]. The hypertension screening survey was administered daily for the first two weeks postpartum , subsequently at three times per week (MWF) for weeks 3 and 4 postpartum, and then once per week for the remainder of the 6-week study period (ie, weeks 5 and 6 postpartum). Figure 1 contains a screenshot from the MOMitor app showing the primary and secondary hypertension screening question as viewed by a participant.

### Outcome Measures

All participants throughout the study period received the following question items for hypertension screening, which asked: (1) “is your blood pressure greater than 140/90?,” and (2) “do you feel dizzy or lightheaded, short of breath, or have a terrible headache?” A participant is considered to have a positive screen for potential hypertension if they answer “yes” to either of the hypertension question items, where both items provide dichotomous ‘yes’ and ‘no’ response options. We define the primary outcome as a positive screen from question 1 and the secondary outcome as a positive screen for question 2. A participant may contribute multiple positive screens throughout the 6-week study period of their enrollment. To clarify, all participants throughout the study period received both questions 1 (the primary outcome for hypertension screening) as well as question 2 (a secondary outcome).

A subset of participants beginning in April 2023, were asked an additional question: “Please report your current Blood Pressure.” Starting in October 2022, another subset of participants were asked, “What is your blood pressure reading?” This question was later split in October 2023 into two separate items: “What is the top number of your blood pressure reading?” and “What is the bottom number of your blood pressure reading?” Numerical values were entered by the participant for BP readings. We classify people with elevated systolic or diastolic BP as a positive screen. Furthermore, we examine the subset of participants who provided these numerical BP readings for concordance with responses that are used to define the primary outcome (ie, ‘yes’ to question 1, indicating a positive screen). Patients with a positive screen received a call from a registered nurse, and their prescriptions and/or clinic visit follow-ups were triaged based on a protocol. The questions requesting numerical input of blood pressure readings were only available at later dates of the study due to technical improvements in the technology used to capture participant responses.

### Other Measures

Potential confounders measured at enrollment included the following sociodemographic and clinical characteristics: age, race, marital status, education, insurance status and type, mode of delivery, total number of pregnancies, and history of diabetes mellitus. Each factor was controlled and selected for inclusion in the analysis based on a literature review of factors related to hypertension risk during postpartum [18,27,28].

### Statistical Analysis

Baseline characteristics at enrollment for all included study participants and for those who completed the hypertension screening in week 6 are described. We describe the proportions of participants who had and did not have positive hypertension screenings for weeks one to six, stratified by type of hypertension previously experienced.

In this study, proportions are defined as the number of individuals with a positive screen divided by the total number of participants with available responses at each time point, noting that the denominator may vary across each data collection interval, reflective of participant responsiveness. For visualization, we plot the proportions of positive hypertension screens across all possible data collection intervals. To provide a weekly summary, we plot proportion of positive screens in each postpartum week.

We then estimate within- and between- participant effects using a generalized linear mixed model for positive hypertension screen for weeks one to six. This model will include both fixed and random effects, controlling for age, race, marital status, education, insurance, mode of delivery, total number of pregnancies, and history of diabetes mellitus. We also estimate within-participant differences between week one and week six using generalized linear mixed models with random and fixed effects for positive hypertension screen, controlling for confounders. We model time flexibly and will require a minimum number of two observations per participant to be included in the primary analysis. We will conduct sensitivity analyses to test whether imputation of missing observations via a last observation carried forward approach is viable. We also examine the last observation (hypertension screening measurement) available per week, in a given week where there are multiple observations, in another sensitivity analysis.

For participants who received the numerical BP questions, we calculated concordance between positive screens from question items 1 and 2 using the subgroup of participants who were prompted to report numerical values of their BP readings. A participant was considered to have a positive screen if their BP exceeded 140/90, or if it exceeded 140 for the top number or 90 for the bottom number. Both the within- and between-participant analyses described above are repeated in this subgroup of positive screens validated by numerical BP readings.

## Results

The study was funded in July of 2021 and data collection was initiated in November of 2021. Data collection for this study has been completed as of June 2025, per protocol, though data continues to be collected for the parent study from which this hypertension assessment component is derived. The parent study assesses a host of physical and mental health conditions among postpartum women with different risk factors and clinical histories. Data analysis is expected to be completed by Spring 2026. Publication of results are planned for Summer of 2026.

## Discussion

Our study will be among the few to evaluate the trajectory of hypertension and blood pressure via remote digital health monitoring in the postpartum period. It will provide insight into whether this approach is associated with detection of hypertension outcomes among postpartum women whose blood pressure was regularly tracked through a smartphone-based system. We hypothesize that hypertension will be detected in some participants throughout the monitoring period via positive screens for high BP and hypertension-related symptoms, but we anticipate that a greater proportion of positive hypertension screens will be detected in the first two weeks postpartum relative to the final weeks of the postpartum monitoring period.

According to ACOG, women with hypertensive disorders of pregnancy should have their BP assessed within 72 hours and again between 7 and 10 days after delivery [9,29]. Prior research also indicates that remote BP monitoring is an acceptable approach to new mothers [29]. Most existing studies on the effectiveness of remote BP monitoring have been randomized clinical trials or feasibility and implementation studies [20]. These investigations found that participation in remote BP monitoring was associated with nearly twice the likelihood of recording at least one BP measurement within 10 days postpartum as accessible to the treating healthcare provider. More than 80% of participants also reported satisfaction with their postpartum care when using home BP monitoring. Additionally, RCTs have demonstrated meaningful improvements in diastolic blood pressure among women with prior gestational diabetes or preeclampsia during the postpartum period [20].

Previous studies have reported reduced hospital readmission rates among women participating in similar remote BP monitoring interventions [30,31], though the trajectory of hypertension positive screens from remote monitoring interventions is not well characterized. This gap is clinically meaningful as the highest risk for adverse hypertension outcomes is in the postpartum period covered by our remote BP monitoring period, where there are also the fewest opportunities for insurance-covered postpartum healthcare visits between the delivery discharge and the routine 6-week follow up visit. Our study will contribute to growing evidence by fully characterizing hypertension trajectories through the postpartum period and we plan to use this data to inform investigation of the value and effectiveness of remote blood pressure monitoring delivered through mobile app by also assessing clinical outcomes from acute hypertension-related events as well as health service use and economic outcomes.

The strengths of the study include a rigorous recruitment strategy, retention strategy to mitigate potential losses to follow up, well-tested smartphone app, robust clinician involvement when potential hypertension is detected (ie, safety monitoring for participants), and a pre-specified analytical protocol to improve transparency. There are limitations to consider, including the potential for selection bias as we recruited only participants with a history of hypertension, such as preeclampsia, eclampsia, or gestational hypertension, or those with a current or prior elevated blood pressure reading (≥140/90), rather than recruitment of all postpartum women presenting for delivery of an infant. Consequently, our findings may not be generalizable to all postpartum women, including those who develop de novo postpartum hypertension. Although hypertension information was self-reported, patients submitted their blood pressure responses in real time, which helps minimize recall-related bias, but this strategy is subject to limitations of bias related to measurement error (ie, if the participant misunderstands the question or enters in their BP readings incorrectly).

Our study will strengthen understanding of blood pressure trajectories among high-risk postpartum women whose blood pressure is frequently monitored through a smartphone app Findings will provide evidence to support whether widespread implementation of this digital health surveillance approach to improve hypertension outcomes in maternal health, especially in the postpartum period, is clinically warranted. Publication of study findings are planned to be disseminated via a scientific journal in the JMIR network.

## Abbreviations

ACOG: American College of Obstetricians and Gynecologists
AHA: American Heart Association
EMA: Ecological Momentary Assessment
HDP: hypertensive disorders of pregnancy
ICD: International Statistical Classification of Diseases
PPHTN: postpartum hypertension
RCT: randomized clinical trial
REDCap: Research Electronic Data Capture
